# Effect of Smartphone Usage on Neck Muscle Endurance, Hand Grip and Pinch Strength among Healthy College Students: A Cross-Sectional Study

**DOI:** 10.3390/ijerph18126290

**Published:** 2021-06-10

**Authors:** Adel Alshahrani, Mohamed Samy Abdrabo, Sobhy M. Aly, Mastour Saeed Alshahrani, Raee S. Alqhtani, Faisal Asiri, Irshad Ahmad

**Affiliations:** 1 Department of Medical Rehabilitation Sciences-Physiotherapy Program, College of Applied Medical Sciences, Najran University, Najran 55461, Saudi Arabia; amsalshahrani@nu.edu.sa (A.A.); msabdrabou@nu.edu.sa (M.S.A.); smali@nu.edu.sa (S.M.A.); rsalhyani@nu.edu.sa (R.S.A.); 2 Department of Basic Sciences, Faculty of Physical Therapy, Cairo University, Cairo 12613, Egypt; 3 Department of Biomechanics, Faculty of Physical Therapy, Cairo University, Cairo 12613, Egypt; 4 Department of Medical Rehabilitation Sciences, College of Applied Medical Sciences, King Khalid University, Abha 62529, Saudi Arabia; msdalshahrani@kku.edu.sa (M.S.A.); iabdulhamed@kku.edu.sa (I.A.)

**Keywords:** smartphone addiction, hand grip, neck muscle endurance, neck flexors, neck extensors

## Abstract

In recent years, there has been a significant increase in global smartphone usage driven by different purposes. This study aimed to explore the effect of smartphone usage on neck muscle (flexors and extensors) endurance, hand grip, and pinch strength among young, healthy college students. In total, 40 male students were recruited for this study; 20 of them belonged to the smartphone-addicted group, while the other 20 were in the non-addicted group based on their smartphone addiction scale—short version (SAS-SV) scores (the threshold for determining smartphone addiction: 31/60). Neck flexor endurance time, the ability to perform a neck extensor muscle endurance test, and hand and pinch grip strength were assessed. Multivariate analysis of variance (MANOVA) was used to assess between-group differences in the mean values of neck flexor endurance time, hand grip, and pinch grip. A significant group effect (Wilks’ lambda = 0.51, F _(5,34)_ = 6.34, *p* = 0.001, partial eta squared = 0.48) was found. A decrease in neck flexor endurance time was observed in the smartphone-addicted group compared with that of the non-addicted group (*p* < 0.001). However, there was no notable difference in the neck extensor muscle endurance test or in hand grip and pinch grip strength of both hands between groups (*p* > 0.05). Using a smartphone for a prolonged time might affect neck flexor muscle endurance; however, more research is needed to explore the long-term effects of using smartphones on neck muscle endurance and hand/pinch grip strength and the risk of developing upper limb neuromusculoskeletal dysfunction.

## 1. Background

Smartphones have become an essential object in everyday life; people of all ages have started using smartphones for different daily tasks. Smartphones are not only a means of communication but also a key source of information and entertainment. The world is becoming increasingly dependent on smartphones, and Saudi Arabia is ranked third in the world for smartphone usage, which can indirectly lead to various musculoskeletal problems [1].

The continuous checking and/or utilization of smartphone applications for prolonged periods is associated with stress, withdrawal, anxiety, sleep disturbance, decreased physical activity, poorer academic performance, and deterioration in wellbeing [2]. Several studies have shown an association between time spent using smartphones and the severity of musculoskeletal complaints [3]. Moreover, studies have reported the harmful impact of smartphones on neck and upper extremity function: the neck becomes more stressed due to the overuse of smartphones, leading to neck muscle weakness [4]. It was reported that the prevalence of neck pain ranges between 17.3% and 67.8% among smartphone users, and the lifetime prevalence is 55.8% [5].

Individuals with minor neck pain who often bend their neck considerably more than their healthy counterparts might have musculoskeletal symptoms due to the prolonged usage of smartphones [3]. Recent research has established that patients with cervical pain show a delay in deep neck flexor (DNF) activation when performing certain tasks with their upper extremity, which denotes a significant deficit in the DNF muscles of the neck that control the cervical spine [6].

Forward head posture or poking chin posture is a poor posture commonly adopted during prolonged smartphone use in a seated position. Aberrant DNF activity with the addictive use of smartphones might cause constant tissue overloading, trauma, and neck pain [7]. Moreover, DNF training has been recommended to optimize cervical spine function [8].

Smartphone use might also affect hand function [9,10,11]. Hand grip strength, best described as the total amount of static force exerted by the hand through squeezing a dynamometer, might be affected by variations in position, such as the elbow positioned in extension more than flexion (90%), forearm support, and standing or sitting during measurement [12,13,14].

Many studies have investigated the psychological, economic, mental, academic, and physical effects of smartphone addiction on university students, but there was a need to study the effects of smartphone addiction among college students in Najran, Saudi Arabia on neuromusculoskeletal dysfunction. Therefore, the objective of this study was to explore the effects of smartphone addiction on neck muscle (flexors and extensors) endurance, hand grip, and pinch strength among healthy college students who used smartphones for various periods of time.

The hypothesis of this study is that there is no difference in the effects of smartphone addiction on neck muscle endurance or hand grip and pinch strength among healthy college students.

## 2. Methods

### 2.1. Study Design and Setting

A cross-sectional study was conducted at the Applied Medical Sciences College, Physiotherapy Clinics, Najran University, Saudi Arabia.

### 2.2. Ethical Approval

Ethical approval was obtained from the institutional review board of Najran University (ref. no.: 01-03-03-2019 EC). A signed informed consent to participate was collected from all students before data collection.

### 2.3. Participants

A total of 40 healthy male students who used smartphones for more than one year, with ages ranging from 18 to 27 years, were recruited from the College of Applied Medical Sciences, Najran University, Saudi Arabia. Students who had neck pain with or without radiating/radicular pain in the upper extremity; congenital deformities in the upper extremity and neck; any history of diagnosed neurological, rheumatic, musculoskeletal, or cardiorespiratory diseases; or previous surgeries on the neck and/or upper limbs were excluded from the study.

The sample required was calculated using G*POWER statistical software (version 3.1.9.2; Universitat Kiel, Germany). Using an α value of 0.05 and a β value of 0.2 to find a large effect size relating to a change in the DNF endurance time between groups, 20 participants in each group were deemed sufficient.

A smartphone addiction scale—short version (SAS-SV) was used to establish the addiction level of smartphone use among students [15]. It contained 10 questions rated using a Likert scale ranging from 1 to 6, with 1 representing ‘strongly disagree’ and 6 ‘strongly agree’. In the literature, the cutoff value for determining smartphone addiction was 31/60 for males and 33/60 for females. Student participants were divided into two groups based on their SAS-SV scores for smartphone addiction. The smartphone-addicted group comprised students with a total score of more than 31, while the non-addicted group included students who scored equal to or less than 31 on the SAS-SV, as shown in Table 1.

### 2.4. Procedure

Demographic data, including age, self-reported hand dominance, hand used to hold/type on the smartphone, duration of smartphone use, and purpose of smartphone use (e.g., browsing, gaming, chatting, writing documents, and social media), were collected from all the students.

The study was carried out at Najran University between September 2020 and January 2021. It was a cross-sectional experimental study design. Participants were randomly distributed between group A (smartphone-addicted) and group B (non-smartphone-addicted). Data were collected during April 2019–July 2019 through a questionnaire to identify addicted and non-addicted participants.

We assessed hand grip strength using a handheld dynamometer, pinch strength using a pinch meter, DNF endurance using a DNF endurance test, and cervical extensor endurance using a cervical extension endurance test (CEET).

### 2.5. Neck Flexor Endurance Assessment

For the DNF endurance test [7,16], the students adopted a hook-lying position with their hands resting on the abdomen. They were asked to tuck in the chin maximally and lift the head around 2.5 cm off the table or the plinth. The students were asked to hold this position isometrically, and the time taken to maintain this position without substitution strategies was recorded. The test was stopped when the skin folds on the neck started to separate, or the subject’s head touched the examiner’s hand placed under the occiput.

### 2.6. Neck Extensor Endurance Assessment

For the CEET [17], the students were instructed to lie prone with their neck and head outside the table or plinth. The ability of students to hold their chin tucked for 60 s while their neck was in a neutral position was evaluated. Only one attempt was made for the neck to complete the CEET to avoid fatigue in the neck muscles. However, the procedure was explained to the participants one day before it took place. Sebastian et al. (2015) interpreted a 5–10 degree change in the position as a positive finding for the weakness of (deep) neck extensors. The test was considered positive if there was an increase in chin-length or an inability to maintain the chin tuck owing to dominant superficial cervical extensor activity. When the patient was not able to hold the starting position (chin tucked), this indicated weakness in both the deep and superficial neck extensors.

### 2.7. Hand Grip and Pinch Strength Assessment

Students performed the grip strength assessment with their dominant hand while sitting. As recommended by the American Society of Hand Therapists’ protocol, they sat in a comfortable chair with the shoulder neutrally rotated and adducted, the elbow flexed at 90°, and the wrist and forearm in a neutral position. A Jamar hand dynamometer and a pinch meter (Sammons Preston, Inc., Bollingbrook, IL, USA) were used to assess grip and pinch strength, respectively [18,19]. The maximum grip and pinch strengths were measured in kilograms (kg), with the participant exerting maximum force to grip or pinch with verbal encouragement. We took an average of three attempts with a one-minute break between each trial.

### 2.8. Data Analysis

The Shapiro–Wilk test was used to test the normal distribution of data, and Levene’s test was used to check whether the groups had equal variances. A between-group comparison of student characteristics was conducted using the Chi-squared test for categorical data and an independent *t*-test for numerical data. Multivariate analysis of variance (MANOVA) was performed to compare the mean values of neck flexor endurance time, hand grip, and pinch grip between both groups. Post-hoc tests using the Bonferroni correction were carried out for subsequent multiple comparisons. The significance level for all statistical tests was set at *p* < 0.05. Statistical analysis was performed using the Statistical Package for Social Studies (SPSS) version 25 for Windows (IBM SPSS, Chicago, IL, USA).

## 3. Results

### 3.1. Participant Characteristics

The characteristics of the students are summarized in Table 1. No significant difference between the groups was observed for mean age, weight, height, and body mass index (BMI) (*p* > 0.05). In addition, there was no substantial variation between the dominant hand and the hand used for holding/typing on smartphones between both groups.

### 3.2. Comparison of Neck Extensor Endurance between the Smartphone-Addicted and Non-Addicted Groups

The students of both groups successfully passed the extensor endurance test by exceeding the 60 s mark.

### 3.3. Comparison of Neck Flexor Endurance Time, Hand Grip, and Pinch Strength between the Smartphone-Addicted and Non-Addicted Groups

There was a notable group effect (Wilks’ lambda = 0.51, F _(5,34)_ = 6.34, *p* = 0.001, partial eta squared = 0.48). Table 2 shows descriptive statistics of the flexor endurance test, hand grip, and pinch grip, as well as the *p* values for between-group comparisons. A decrease in flexor endurance time was observed in the smartphone-addicted group compared with that of the non-addicted group (*p* < 0.001). However, no significant difference in hand grip or the pinch grip strength of both hands was found between the groups (*p* > 0.05).

## 4. Discussion

This study investigated the effect of smartphone use on hand grip strength, pinch strength, and neck muscle (flexors and extensors) endurance among healthy college students. The findings of this research revealed no significant variation in hand grip strength, pinch strength, or neck extensor endurance for smartphone-addicted and non-addicted students. However, a substantial decrease in neck flexor endurance time in the smartphone-addicted students was observed when compared with that of the non-addicted students.

Our study results revealed that flexor endurance time (s) was significantly lower in the smartphone-addicted group in comparison with those in the non-addicted group (*p* < 0.001). Consistent with these results, Kim et al. (2013) found that among 18 healthy smartphone users, the duration of smartphone use had an influence on the neck and back flexion angles [20]. Domenech et al. (2011) concluded that the average DNF endurance test time was 39 s for males and 29 s for females between the ages of 20 and 80 years, and the interrater reliability for DNF muscle endurance testing was 66% [14].

Flexing the neck is the most usual and regular posture adopted by mobile phone users; they tend to adopt this posture when looking at their devices for long periods. The length of time devoted to smartphone usage and adopting flexed neck postures may cause pain and discomfort to the neck region in the long term [21]. Furthermore, smaller smartphone screen sizes might indirectly influence the adoption of different neck positions to enhance looking at the screen. Smaller screens might cause more forward bending of the neck, reducing the distance between the screen and the user’s eyes [22]. A compensatory forward neck bending may be occasioned with increased neck muscle activity, which might have affected DNF endurance in the smartphone-addicted group.

Our study found no significant difference in the CEET between the smartphone-addicted and non-addicted groups since our participants were all asymptomatic. Moreover, Halvorsen et al. (2014) found that asymptomatic participants did very well when compared to participants who experienced neck pain [23]. Sebastian et al. (2015) also found that most of their participants with neck pain were unable to complete the test [17]. Whether our asymptomatic students, especially the smartphone-addicted group, will develop neck pain and show poor endurance of the neck extensors in the long term warrants further investigation.

The results of this study revealed that hand grip and pinch grip strength were not affected by prolonged smartphone use, which may be explained by the fact that most participants were young, as they were recruited from university students who engaged in similar hand activities (such as computer use and writing) that demand repeated static muscle contractions, which maintain strength in the hand muscles. Our finding is similar to the finding of Inal et al. (2015). They concluded that SAS scores were negatively correlated with grip and pinch strength, but the correlations reported were weak (r = −0.110 and r = −0.281, respectively) [9], which supports the findings of our study, as subjects with higher SAS scores in the addicted group had lower grip and pinch strength (21.85 ± 5.2 kg and 4.25 ± 1.11 kg, respectively) than those in the non-addicted group (24.4 ± 4.82 kg and 4.6 ± 1.09 kg, respectively). Furthermore, our results for hand grip and pinch strength may be attributed to the fact that 75% of participants reported using their smartphone with the dominant hand and 25% with both hands in the current study. However, another study showed some correlation between hand grip and smartphone usage duration [10,24,25]. This might be because we did not ask the participants to use their smartphones during the period when we collected the data. We wanted to examine the impact of addiction score on hand and grip strength in real life, not for prolonged smartphone usage.

In our present research, no major difference in hand grip strength and pinch strength was observed because our participants were free from neck pain. Fayez et al. (2014) studied grip strength on dentists with chronic neck pain using a dynamometer, and they found a significant difference in grip strength [12]. These findings may be explained by abnormality of the sensory–motor integration, especially in the neck, leading to pain and aberrant motor control and strength. On the contrary, Huysmans et al. (2008) discovered that participants with neck pain had considerably greater grip strength compared to those participants without pain [26]. Further studies are warranted to substantiate the effect of smartphone use on hand grip strength.

Cervical flexion is the most regular posture adopted while using a smartphone for an extended period, and this might lead to musculoskeletal problems [27,28] in the cervical region [28].

Several studies found that more than 80% of mechanical steadiness in the cervical region is achieved by the deep neck muscles; functionally, longus capitis and longus colli are deep neck flexors that provide longitudinal dynamic cervical spine stability [16]. Other studies have indicated that weakness in these muscles is due to chronic neck pain [29,30,31]. Although the male students in the smartphone-addicted group of our study showed decreased DNF endurance, they had not developed neck pain at the time of the study. However, a longitudinal study is required to monitor their musculoskeletal symptoms in the long term.

The present research demonstrated that excessive use of a smartphone and adopting a flexed cervical spine posture are associated with high addiction, which can cumulatively pose a long-term health issue. Therefore, awareness should be raised among students about the long-term ill effects of smartphone addiction.

We acknowledge several limitations in our study. This study is a preliminary one for a future research project involving more than one geographical location. In future investigations, we recommend including participants of all age groups, and cohort studies can be conducted to rule out the long-term effects of smartphone addiction. Furthermore, considering the cross-sectional design of the study, a cause-and-effect relationship cannot be established between smartphone addiction and DNF endurance. Lastly, the lack of detailed information on how, when, and why our population utilized their phones might have affected the results of our study. Future studies need to collect more information on individuals’ habits, perceptions, and purposes driving their smartphone usage and addiction behavior.

## 5. Conclusions

The findings of this study showed that smartphone addiction can negatively affect neck flexor endurance, but not hand grip and pinch strength, in young, healthy male college students. Therefore, further studies could investigate whether reducing the duration of smartphone usage mitigates the negative effect of smartphone overuse on neck flexor endurance.

## Figures and Tables

**Table 1:** Between-group comparison of participants’ characteristics.

	Smartphone-Addicted Group	Non-Smartphone-Addicted Group	*p* Value
	Mean ± SD	Mean ± SD	
Age (years)	21.8 ± 1.6	22.3 ± 1.45	0.30
Weight (kg)	69.2 ± 7	67.5 ± 7.97	0.47
Height (cm)	173.25 ± 3.2	174.05 ± 4.65	0.53
BMI (kg/m²)	23.04 ± 2.11	22.37 ± 3.34	0.45
SAS-SV scores	37.35 ± 3.23	27.2 ± 2.3	0.001
Dominant hand			
Right	18 (90%)	19 (95%)	0.54
Left	2 (10%)	1 (5%)	
Preferred hand for smartphone use			
Dominant hand	13 (65%)	17 (85%)	0.34
Both hands	7 (35%)	3 (15%)	

$\bar{x}$, mean; SD, standard deviation; *p* value, level of significance.

**Table 2:** Between-group comparisons of neck flexor endurance time, hand grip, and pinch strength.

	Smartphone-Addicted Group	Non-Smartphone-Addicted Group		
	Mean ± SD	Mean ± SD	MD (95% CI)	*p* Value
Flexor endurance time (s)	14.45 ± 3.95	20.3 ± 2.84	−5.85 (−8.05, −3.64)	0.001
Hand grip strength (kg)				
Dominant hand	21.85 ± 5.2	24.4 ± 4.82	−2.55 (−5.76, 0.66)	0.11
Non-dominant hand	20.05 ±4.97	21.35 ± 3.82	−1.3 (−4.14, 1.54)	0.36
Pinch grip strength (kg)				
Dominant hand	4.25 ± 1.11	4.6 ± 1.09	−0.35 (−1.05, 0.35)	0.32
Non-dominant hand	3.45 ± 0.75	3.65 ± 0.93	−0.2 (−0.74, 0.35)	0.46

$\bar{x}$ mean; SD, standard deviation; MD, mean difference; CI, confidence interval; *p* value, level of significance.

## Data Availability

The datasets used and/or analyzed during the current study are available from the corresponding author on reasonable request.

## Abbreviations

CEET: cervical extension endurance test; DNF: deep neck flexors; MANOVA: *multivariate analysis of variance;* SAS-SV: smartphone addiction scale—short version.
